# Early premorbid of children with ASD

**DOI:** 10.1192/j.eurpsy.2021.552

**Published:** 2021-08-13

**Authors:** M. Kalinina, G. Shimonova

**Affiliations:** 1 Child Psychiatry, MHRC RAMS, Moscow, Russian Federation; 2 Child Psichiatry, MHRC RAMS, Moscow, Russian Federation

**Keywords:** early childhood, autism, high risk group, a prospective study, autism, early childhood

## Abstract

**Introduction:**

According to numerous studies, the origins of most endogenous mental diseases, in particular, autism, lie in early childhood. This concept is based on the classical theories of diathesis.

**Objectives:**

The purpose of this study was to clarify the phenomenology of mental disorders in children with an assessment of the prognostic significance of symptoms that preceded the development of the disease for future mental health.

**Methods:**

The clinical material was collected during the survey of a child population. Selected for prospective observation was 40 children (1-3 years old) from the high-risk group for schizophrenia with functional disorders of the endogenous spectrum. All patients were examined by clinical methods and pathopsychological, neurophysiological. Psychometric scales PANSS, CARS were used. The results were mathematically evaluated using the Statistica 7 program.

**Results:**

The clinical picture of the mental state of young children, in children with autistic disorders in 1,5-3 years, was determined by a specific complex of disorders, which were reduced to a general deficit, especially in the emotional sphere, vegetative dysregulation, most often, the sleep-Wake rhythm. Motor skills, as a rule, did not lag significantly behind the age standards. The onset of actual autistic disorders was noted older than 1 year of life. Сhildren received medication and corrective therapy. The detailed clinical picture of violations developed gradually. Dynamics of psychopathological picture in (80,0%) children was regressive.

**Conclusions:**

The study shows the importance of preventive measures in people related to ASD, sparing individual approach in education and therapy.

